# Supplementary material for: Analysis of individual differences in neurofeedback training illuminates successful self-regulation of the dopaminergic midbrain
Source: Commun Biol. 2022 Aug 19;5:845. doi: 10.1038/s42003-022-03756-4 (PMC9391365; doi:10.1038/s42003-022-03756-4)
Supplement: Supplementary file 2 — Supplementary Information [file 42003_2022_3756_MOESM2_ESM.pdf]

Supplemental Material on "Individual differences in neurofeedback training illuminates successful self-regulation of the dopaminergic midbrain"

Supplementary Figure 1: Distribution of midbrain DRT 2
Supplementary Figure 2: Correlation midbrain DRT and slope of SN/VTA signal change during training 2
Supplementary Table 1: Cognitive control network analysis of correlation between midbrain DRT and non-midbrain DRT in standard feedback group 3
Supplementary Table 2: Correlation between midbrain DRT and non-midbrain DRT in inverted feedback group 4
Supplementary Table 3: Disjunction analysis of standard and inverted feedback groups 5
Supplementary Table 4: Conjunction analysis of standard and inverted feedback groups 6
Supplementary Figure 3: Scheme for calculation of temporal difference error 7
Supplementary Table 5: Decreasing relation to SN/VTA temporal difference coding 8
Supplementary Figure 4: Relation of dlPFC activity to SN/VTA temporal difference error coding in early and late neurofeedback training phase 9
Supplementary Table 6: Reward adaptation and general reward sensitivity in MID task correlated with midbrain DRT 10
Supplementary Note 1: Instructions in different studies 10
MacInnes and colleagues (2016) 10
Sulzer and colleagues (2013) 10
Kirschner and colleagues (2018) 11
Comparison 11
Supplementary Figure 5: Neurosynth network terms 12
Supplementary Note 2: Control analysis for spatial specificity of dopaminergic midbrain regulation 13
Supplementary Figure 7: MID task trial structure 14
Supplementary Table 8: Strategies used 14
Supplementary Note 3: Dynamic Causal Modelling analysis 15
Supplementary Table 10: Confidence interval testing by bootstrapping 17
Supplementary Note 4: Validation analysis of conditions *IMAGINE_REWARD* and *REST* as separate conditions 18

Supplementary Figure 1: Distribution of midbrain DRT

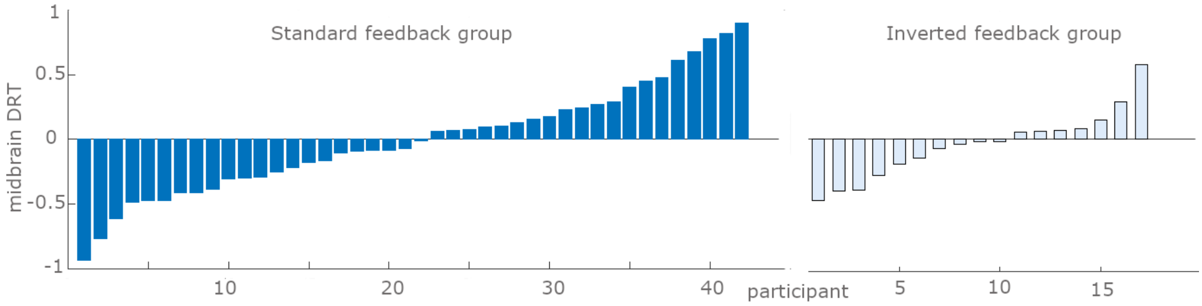

Supplementary Figure 1: Distribution of midbrain DRT measure in standard feedback group and inverted (control) feedback group

Supplementary Figure 2: Correlation midbrain DRT and slope of SN/VTA signal change during training

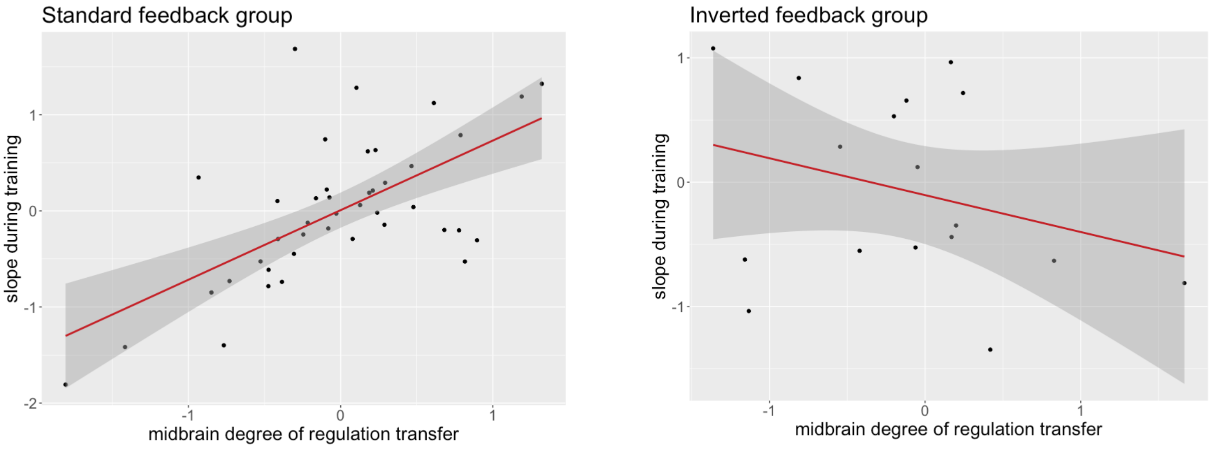

Supplementary Figure 2: Positive correlation between the slope of SN/VTA signal change during neurofeedback training and midbrain DRT for the standard feedback group, but not for the control group *(standard neurofeedback group $\rho = 0.62$, $p < 0.001$, n = 42 subjects; inverted feedback group $\rho = -0.3$, $p = 0.25$, n = 17 subjects, one-sided test for difference between correlations in independent samples: $z = 3.03$, $p = .001$). The grey shaded area identifies the 95 % confidence interval.*

Supplementary Table 1: Cognitive control network analysis of correlation between midbrain DRT and non-midbrain DRT in standard feedback group

***Supplementary Table 1**: Overlap of cognitive control template with regions showing significant correlation between midbrain and non-midbrain DRT in standard feedback group*

			MNI Coordinates		
**Region Label**	**# voxels**	**t-value**	**x**	**y**	**z**
Middle Frontal Gyrus (dorsolateral prefrontal Cortex)	436	4.61	45	31	19
Left Thalamus	536	4.47	-9	-24	10
Temporal Occipital Fusiform Cortex	701	5.85	32	-46	-22
Occipital Fusiform Gyrus	701	4.92	29	-66	-12
Occipital Fusiform Gyrus	562	4.87	-42	-70	-19
Right Cerebral White Matter (Right Middle Temporal Gyrus)	122	4.95	56	-45	-4
Left Caudate	138	5.78	-12	10	1
Middle Frontal Gyrus	151	5.35	-39	5	53
Right Cerebral White Matter (Insula)	65	3.67	27	20	-7
Middle Frontal Gyrus	90	4.54	-42	17	40
Right Thalamus	160	4.40	5	-13	13
Left Cerebral White Matter	152	4.30	-45	-45	1
Frontal Pole	145	4.15	24	59	7
Precentral Gyrus	169	4.07	-41	-9	62
Lateral Occipital Cortex, superior division	64	4.04	39	-61	46
Lateral Occipital Cortex, superior division	109	3.96	27	-64	52
Lateral Occipital Cortex, superior division	114	3.96	-33	-70	50
Precuneus	61	3.91	0	-70	46
Right Cerebral White Matter (dorsolateral prefrontal Cortex)	89	3.88	36	16	37
Frontal Orbital Cortex	80	3.87	-36	22	-6
Right Cerebral White Matter (Superior Temporal Gyrus)	69	3.79	51	-30	7
Right Cerebral White Matter (Right Caudate Nucleus)	68	3.76	14	10	-1
Right Cerebral White Matter (Superior Temporal Gyrus)	69	3.79	51	-30	7
Right Cerebral White Matter (Right Caudate Nucleus)	68	3.76	14	10	-1
Right Pallidum	42	3.72	20	-4	1

For all clusters within cognitive control template based on $t > 3.10$; $p < 0.001$ uncorrected; df = 40; minimum extent = 40; Table shows all local maxima separated by more than 20 mm. Regions were labeled using the Harvard-Oxford atlas and/or the Anatomy Toolbox in parentheses; x,y,z = Montreal Neurological Institute (MNI) coordinates in the left-right, anterior-posterior, and inferior-superior dimensions, respectively.

Supplementary Table 2: Correlation between midbrain DRT and non-midbrain DRT in inverted feedback group

***Supplementary Table 2**: Regions showing significant correlation between mibrain and non-midbrain DRT in inverted feedback group*

			MNI Coordinates		
**Region Label**	**# voxels**	**t-value**	**x**	**y**	**z**
Left Cerebral White Matter (Temporal Lobe)	68	9.49	-23	-25	-1
Left Cerebral White Matter	93	13.3	-14	-49	13
Frontal Orbital Cortex (L IFG)	71	8.38	-29	16	-21
Left Thalamus	332	7.69	-3	-24	-4
Right Thalamus	332	6.10	18	-27	1
Left Cerebral White Matter (Frontal Lobe)	367	7.65	-29	13	19
Left Cerebral White Matter (Frontal Lobe)	174	7.09	-18	46	5
Left Amygdala	51	6.34	-26	-4	-21
Left Cerebral White Matter (L IFG)	125	6.33	-42	29	8
Location not in atlas (Temporal Lobe)	173	6.03	-5	-13	-10
Insular Cortex	56	6.02	-45	5	-9
Lateral Occipital Cortex, superior division	63	6.01	-12	-72	59
Left Cerebral White Matter (Temporal Lobe)	65	5.92	-32	2	-25
Left Cerebral White Matter (Occipital Lobe)	151	5.77	-36	-63	16
Parahippocampal Gyrus, anterior division (L Fusiform Gyrus)	137	5.72	-36	-12	-27
Left Cerebral White Matter (L Parahippocampal Gyrus)	136	5.69	-24	-34	-15
Left Cerebral White Matter (Temporal Lobe)	53	5.25	-42	-36	4
Frontal Orbital Cortex (L IFG)	63	5.03	-33	26	-12
Superior Temporal Gyrus, anterior division	114	4.98	-54	-4	-10
Left Cerebral White Matter (Temporal Lobe)	56	4.83	-45	-39	8
Temporal Pole	50	4.44	-39	1	-43
Inferior Temporal Gyrus	43	4.58	-48	-40	-19

For all clusters, $t > 3.70$; $p < 0.001$ uncorrected; df = 14; minimum extent = 40; Table shows all local maxima separated by more than 20 mm. Regions were labeled using the HarvardOxford atlas and/or the Anatomy Toolbox in parentheses; x,y,z =Montreal Neurological Institute (MNI) coordinates in the left-right, anterior-posterior, and inferior-superior dimensions, respectively.

Supplementary Table 3: Disjunction analysis of standard and inverted feedback groups

***Supplementary Table 3**: Disjunction analysis, reflecting preferential correlation between midbrain and non-midbrain DRT in standard group (Supplementary Table 1) or control group (Supplementary Table 2)*

			MNI Coordinates		
**Region Label**	**# voxels**	**t-value**	**x**	**y**	**z**
Left Cerebral White Matter (L Precuneus)	87	13.31**	-14	-49	13
Left Cerebral White Matter (Temporal Lobe)	62	9.49**	-23	-25	-1
Frontal Orbital Cortex (L IFG)	64	8.38**	-29	16	-21
Left Thalamus	257	7.69**	-3	-24	-4
Right Thalamus	257	6.10**	18	-27	1
Left Cerebral White Matter (Frontal Lobe)	333	7.65	-29	13	19
Left Cerebral White Matter (Frontal Lobe)	88	7.09	-18	46	5
Left Amygdala	48	6.34**	-26	-4	-21
Left Cerebral White Matter (L IFG)	91	6.33**	-42	29	8
Insular Cortex (Temporal Pole)	48	6.02**	-45	5	-9
Lateral Occipital Cortex, superior division	55	6.01	-12	-72	59
Left Cerebral White Matter (Temporal Lobe)	57	5.3	-32	2	-25
Left Cerebral White Matter (Occipital Lobe)	132	5.77	-36	-63	16
Parahippocampal Gyrus, anterior division	122	5.72**	-36	-12	-27
Left Cerebral White Matter (Frontal Lobe)	65	5.54	-9	37	8
Frontal Orbital Cortex (L IFG)	54	5.03**	-33	26	-12
Superior Temporal Gyrus	87	4.98**	-54	-4	-10
Temporal Pole	42	4.43	-39	1	-43

For all clusters, t > 3.10; p < 0.001 uncorrected; minimum extent = 40; Table shows all local maxima separated by more than 20 mm. Regions were labeled using the HarvardOxford atlas and/or the Anatomy Toolbox in parentheses; x,y,z =Montreal Neurological Institute (MNI) coordinates in the left-right, anterior-posterior, and inferior-superior dimensions, respectively.
** Significant difference in direct comparison between veridical and inverted feedback groups (p <.001).

Supplementary Table 4: Conjunction analysis of standard and inverted feedback groups

***Supplementary Table 4**: Conjunction analysis, reflecting common correlation between midbrain and non-midbrain DRT in standard (Supplementary Table 1) and inverted feedback group (Supplementary Table 2)*

			MNI Coordinates		
**Region Label**	**# voxels**	**t-value**	**x**	**y**	**z**
Temporal Occipital Fusiform Cortex	28	5.81	32	-46	-24
Right Cerebral White Matter	154	5.80	5	-16	-10
Right Cerebral White Matter (Right Hippocampus)	32	5.26	21	-18	-10
Left Thalamus	45	5.1	-8	-16	-1
Inferior Temporal Gyrus, posterior division	85	4.20	-60	-40	-16
Parahippocampal Gyrus (Fusiform Gyrus)	32	3.93	-21	-34	-18
Parahippocampal Gyrus, posterior division	25	3.86	-15	-45	-16

For all clusters, $t > 3.10$; $p < 0.001$ uncorrected; minimum extent = 20 (due to conjunction of two contrasts); Table shows all local maxima separated by more than 20 mm. Regions were labeled using the HarvardOxford atlas and/or the Anatomy Toolbox in parentheses; x,y,z =Montreal Neurological Institute (MNI) coordinates in the left-right, anterior-posterior, and inferior-superior dimensions, respectively.

Supplementary Figure 3: Scheme for calculation of temporal difference error

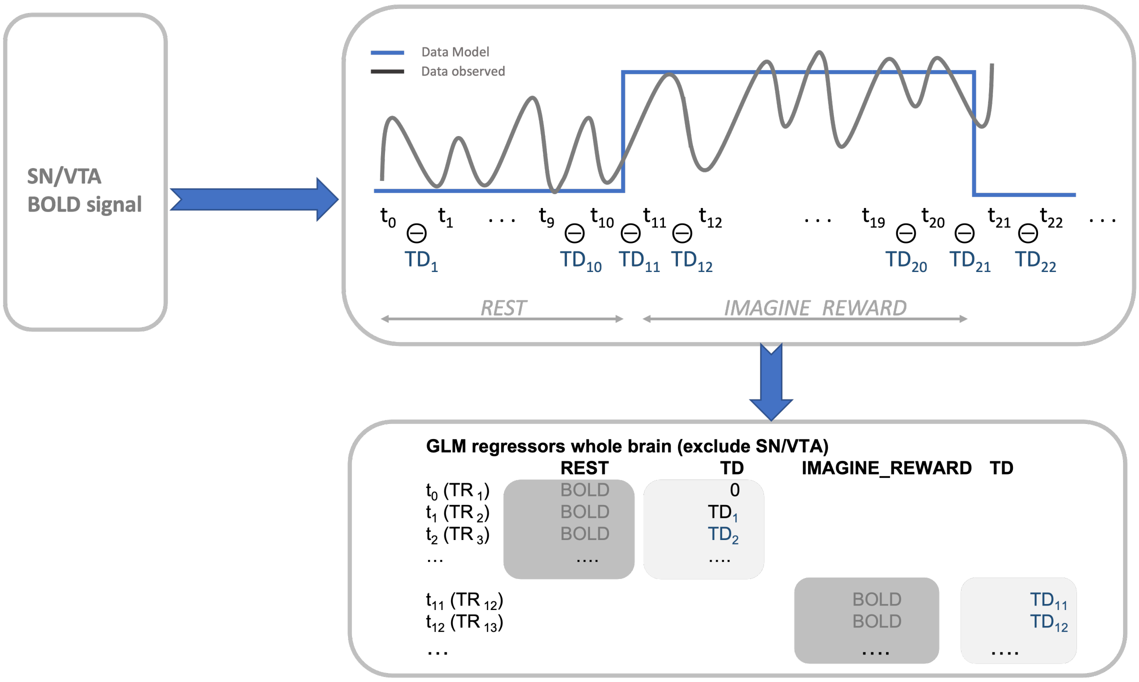

Supplementary Figure 3: Calculation of TR-by-TR temporal difference error: *To calculate temporal difference (TD) errors during neurofeedback training runs, the SN/VTA signal in each single timestep t_i is subtracted from the signal at the previous timestep t_{i-1}, such that TDs were calculated for each TR of the neurofeedback training runs. Thus, the differences t_1 - t_0 , … , t_{22} - t_{21} correspond to the magnitude of TD_1, … , TD_{22}. Shown is one block within a run, comprising the two conditions, REST and IMAGINE_REWARD. In the GLM, the TD values entered as parametric modulator for the BOLD regressors REST and IMAGINE_REWARD at each TR. We interrogated this parametric modulator at the whole-brain level to localize regions where activity correlated with TDs other than SN/VTA. Finally, to identify regions that showed reduced coding of TDs as learning progressed, we contrasted the statistical maps of the second neurofeedback training run with the first training run. Thus, TDs were calculated as TR-wise time-resolved signal, and the reduction in strength of TD coding was assessed run-wise.*

Supplementary Table 5: Decreasing relation to SN/VTA temporal difference coding

Supplementary Table 5*: Cluster table for decreasing relation to midbrain temporal difference error signals during neurofeedback training based on contrast (IMAGINE_REWARD-REST)training_run2 – (IMAGINE_REWARD-REST)training_run1*

			MNI Coordinates		
**Region Label**	**# voxels**	**t-value**	**x**	**y**	**z**
Lateral Occipital Cortex, inferior division	197	4.847	-45	-69	-10
Postcentral Gyrus	210	4.511	50	-25	44
Right Cerebral White Matter (dorsolateral prefrontal cortex)	23	3.393	35	11	37
Right Cerebral White Matter	49	4.357	30	-21	49
Superior Temporal Gyrus, posterior division	36	4.241	-68	-40	13
Left Cerebral White Matter (Left Inferior Frontal Gyrus)	55	4.083	-42	-57	-3
Left Cerebral White Matter (Left Caudate Nucleus)	31	4.072	-18	-4	28
Right Cerebral White Matter (Right Inferior Occipital gyrus)	39	4.068	39	-73	-3
Postcentral Gyrus	68	4.027	-41	-27	49
Superior Parietal Lobule	29	4.010	14	-51	71
Right Cerebral White Matter	30	3.972	39	-37	31
Superior Parietal Lobule	53	3.862	-39	-40	47
Right Cerebral White Matter	36	3.838	29	-48	46
Precentral Gyrus	26	3.827	0	-22	53
Left Cerebral White Matter	34	3.820	-12	-88	8
Supracalcarine Cortex	61	3.758	24	-61	19
Juxtapositional Lobule Cortex (formerly Supplementary Motor Cortex)	21	3.758	-3	-3	47
Lateral Occipital Cortex (Precuneus)	56	3.751	14	-61	59
Lateral Occipital Cortex	72	3.561	-20	-73	52
Lateral Occipital Cortex	24	3.559	-26	-76	38
Substantia nigra/ventral tegmental area	62	4.129	6	-7	-21

For all clusters, t > 3.1; p < 0.001 uncorrected; df = 40; minimum extent = 20; Table shows all local maxima separated by more than 20 mm. Regions were labeled using the HarvardOxford atlas and/or the Anatomy Toolbox in parentheses. x,y,z =Montreal Neurological Institute (MNI) coordinates in the left-right, anterior-posterior, and inferior-superior dimensions, respectively. Note: we report the results for dopaminergic midbrain here because the data for the temporal difference analysis (training runs) are independent from the DRT analysis (baseline and transfer run). However, the temporal difference modulator itself is defined by BOLD activity of the SN/VTA over the training runs.

Supplementary Figure 4: Relation of dlPFC activity to SN/VTA temporal difference error coding in early and late neurofeedback training phase

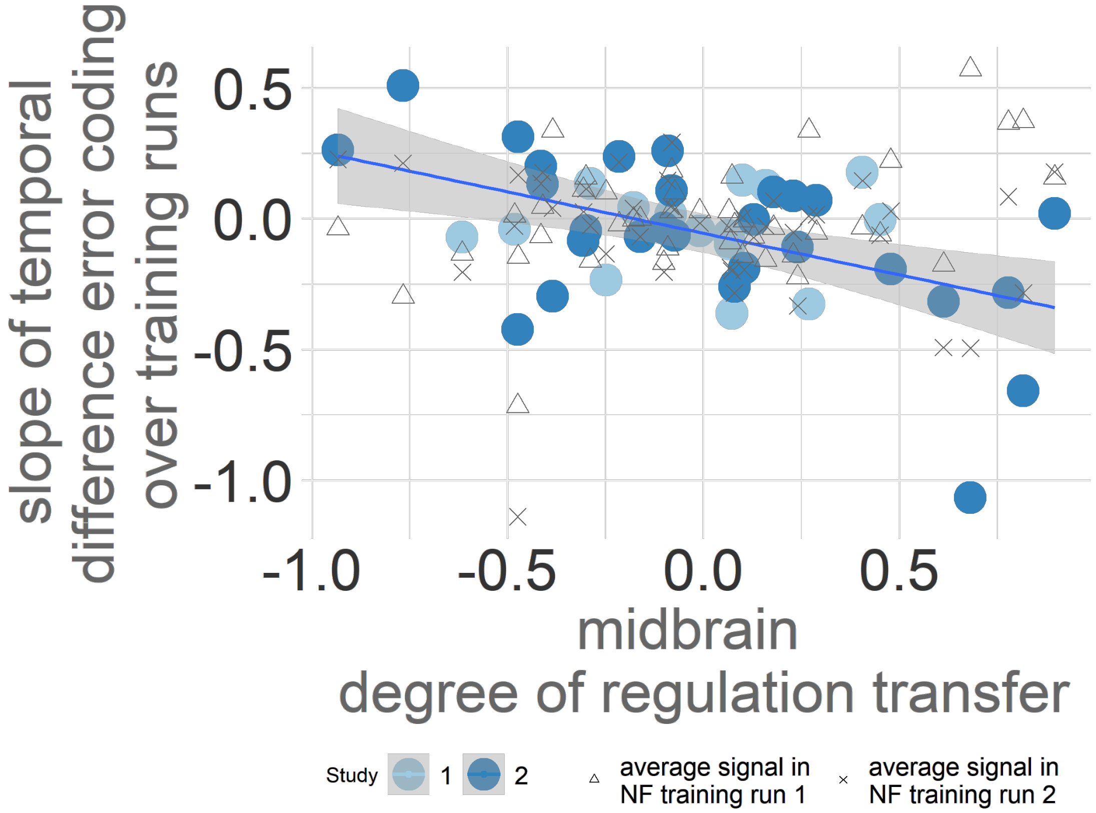

Supplementary Figure 4: *Run-wise relation between SN/VTA temporal difference error coding and dlPFC activity during NF training as function of SN/VTA self-regulation success. The slope of the temporal difference signal in dlPFC over the training runs, corresponding to the parametric difference between the current and immediately preceding feedback activity from the SN/VTA, decreased for the successful participants only. Here, the difference between the late training phase and early training phase is replotted from Figure 4. Moreover, the contrast estimate from the parametric temporal difference modulator is illustrated separately for first and second run, showing decrease of relation to temporal difference error coding in late training for successful regulators. This analysis is based on n = 42 subjects. The grey shaded area identifies the 95 % confidence interval.*

Supplementary Table 6: Reward adaptation and general reward sensitivity in MID task correlated with midbrain DRT

***Supplementary Table 6**: Cluster table for correlation of midbrain DRT with MID task-based reward adaptation (difference of small minus large reward parametric modulators) and general reward sensitivity (sum of small and large reward parametric modulators) as conjunction of all three analysis*

			MNI Coordinates		
**Region Label**	**# voxels**	**t-value**	**x**	**y**	**z**
Middle Frontal Gyrus (dorsolateral prefrontal cortex)	21	3.72	39	8	39
Temporal Occipital Fusiform Cortex	37	4.98	32	-43	-21
Middle Temporal Gyrus, temporooccipital part	344	4.60	-63	-48	-4
Left Cerebral White Matter	32	4.30	-45	-45	1
Frontal Orbital Cortex	64	4.27	-51	26	-13
Right Thalamus	24	4.77	18	-28	-4
Left Cerebral White Matter	23	4.12	-8	-16	-6
Superior Frontal Gyrus	38	3.86	-2	37	58
Parahippocampal Gyrus	22	3.71	20	-12	-28

For all clusters, $t > 3.1$; $p < 0.001$ uncorrected; df = 40; minimum extent = 20 (due to conjunction); Table shows all local maxima separated by more than 20 mm. Regions were labeled using the HarvardOxford atlas and /or the AnatomyToolbox in parentheses; x,y,z =Montreal Neurological Institute (MNI) coordinates in the left-right, anterior-posterior, and inferior-superior dimensions, respectively.

Supplementary Note 1: Instructions in different studies

MacInnes and colleagues (2016)

Available from: https://www.cell.com/cms/10.1016/j.neuron.2016.02.002/attachment/ddbc338c-f251-404b-b648-41e7e6bd64a1/mmc1.pdf, page 10

The participants were instructed to encourage themselves to change a thermometer level according to instructions on the screen.

Sulzer and colleagues (2013)

Available from http://www.ncbi.nlm.nih.gov/pubmed/23791838, page 818 in section Instructions

The participants were instructed to imagine rewarding stimuli to change a moving ball according to the instructions. They were also informed about the 5 s delay of HRF delay.

Kirschner and colleagues (2018)

Available from https://www.thelancet.com/journals/ebiom/article/PIIS2352-3964(18)30472-9/fulltext, page 491 section 3.1

In addition to instructions as given by Sulzer and colleagues, a list of potentially rewarding stimuli was presented with additional ranking of speed to generate such thoughts, vividness and detailedness. The best ranked strategies were suggested to be used in the scanner.

Comparison

The original instructions used by Kirschner and colleagues were given in German. In addition to the summary from the papers above, participants were explicitly instructed to change their thoughts between the regulation trials and figure out successful ones. This is similar to the instructions used by MacInnes and colleagues. Also, all instructions explicitly explained the delay in the feedback signal of several seconds and all studies asked questions about the perceived self-performance afterwards.

The main difference between the instructions used by the three studies concerns the suggested choice of mental strategies – while MacInnes and colleagues focus on self-motivation for the neurofeedback task, the other two studies refer to positive self-memories. Other differences arise from 1) MacInnes and colleagues instructing participants not only about the display of their current performance but also of their average performance and targeted performance and 2) Sulzer and colleagues and Kirschner and colleagues explicitly instructing participants to rest during “Rest” and only imagine positive memories during “Happy Time”.

Supplementary Figure 5: Neurosynth network terms

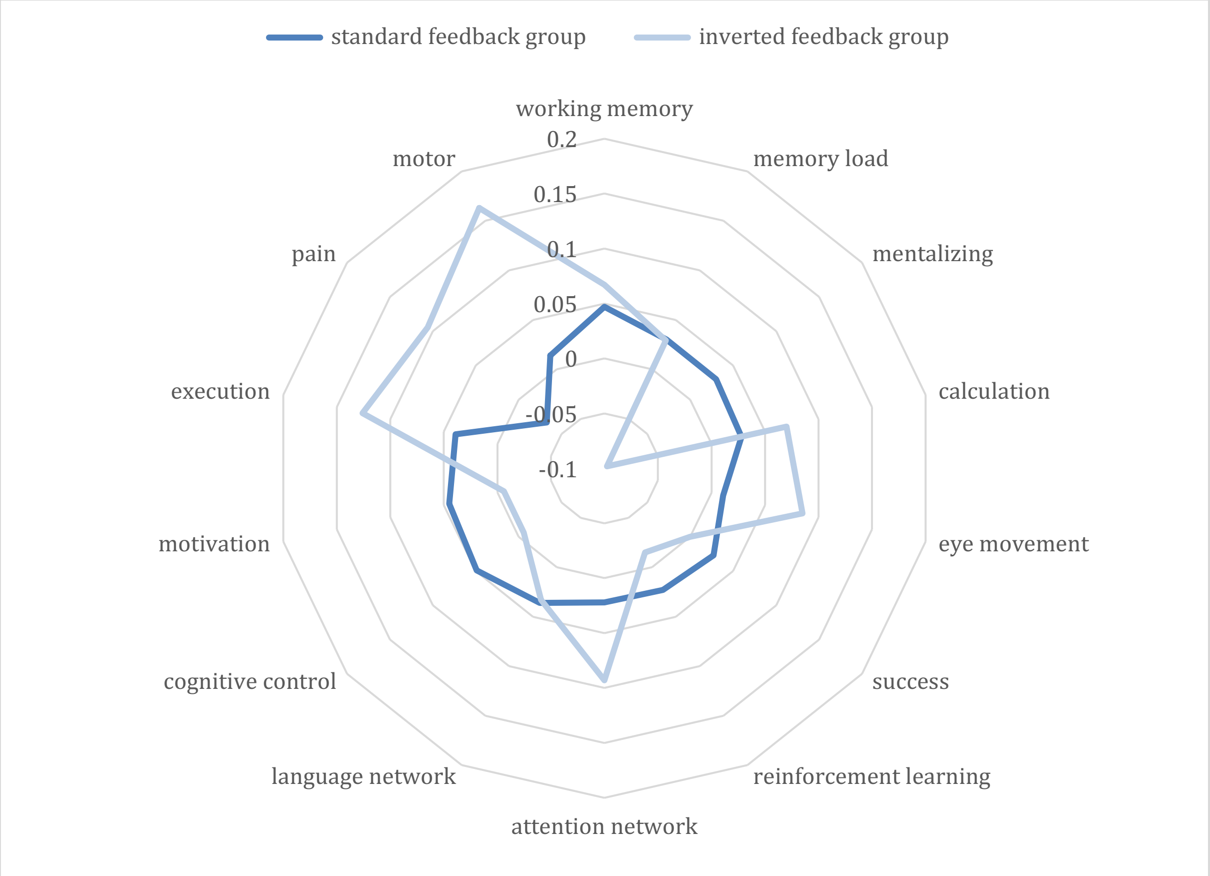

Supplementary Figure 5: Neurosynth database decoding*: To test the functional specificity of our results, we performed a meta-analytic functional decoding analysis using the Neurosynth database (neurosynth.org). We evaluated the representational similarity between the neural patterns of the two resulting maps of the standard feedback group and the inverted feedback group. We selected thirteen different terms in domains covering task-related keywords. Results show that the neural signatures of cognitive control (and motivation) are more similar to non-midbrain DRT than other task-related neural patterns for the standard feedback group. In contrast, for the inverted feedback group the strongest similarities concerned motor functions, task execution and pain. These data support the notion that successful regulation transfer relied on different mechanisms in the two groups. Values on the spider plot represent Pearson's correlation coefficients.*

Supplementary Note 2: Control analysis for spatial specificity of dopaminergic midbrain regulation

To investigate the spatial specificity of our analysis of dopaminergic midbrain self-regulation, we performed the same analysis as described in the main text for SN/VTA but using the neighboring brain region of the parahippocampus as control ROI. This target area is also active during the self-regulation task because the participants perform memory-based strategies. We extracted the parahippocampus mask from the human Talairach atlas in the WFU Pickatlas and performed the identical main effects analysis as described in the main paper. To assess commonalities for the two target ROIs, SN/VTA and parahippocampus, we performed a conjunction analysis based on unthresholded contrast images. This analysis revealed two common areas within the cerebellum and the temporal gyrus (Supplementary Figure 6 and Supplementary Table 7). The limited commonalities between these two target ROIs, especially in striatal and prefrontal areas, are compatible with spatial specificity of our findings using the SN/VTA as target region. To corroborate this interpretation, we also directly compared the results of the two target ROIs in a second level analysis (SN/VTA - parahippocampus) and calculated the conjunction of this difference with the SN/VTA results only. The results of this conjunction analysis revealed similar neural activity patterns as the SN/VTA pattern only. This analysis underpins the local specificity of our results because the interpretation of the results remains the same even when the parahippocampus result pattern has been subtracted.

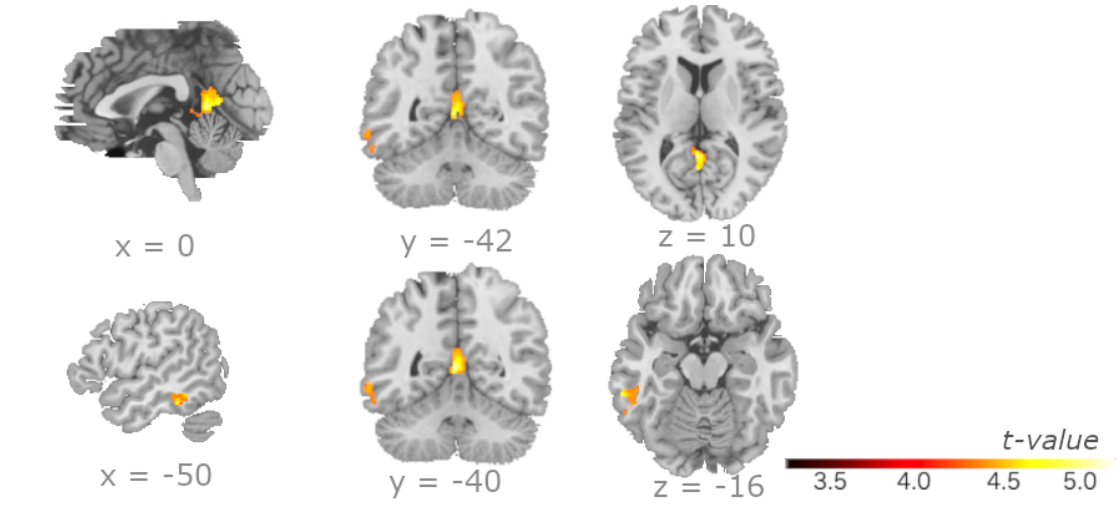

Supplementary Figure 6 Spatial specificity of SN/VTA findings: *Conjunction analysis of two different ROIs – our target ROI SN/VTA and control ROI in parahippocampus – for the main effect analysis of individual regulation transfer revealed little commonalities between these two target ROIs in striatal and prefrontal areas. This is in keeping with spatial specificity of our SN/VTA findings. This analysis is based on n = 42 subjects.*

Supplementary Table 7: *Clusters overlapping in main (coactivation with SN/VTA) and spatial control (coactivation with parahippocampus) analysis*

			MNI Coordinates		
**Region Label**	**# voxels**	**t-value**	**x**	**y**	**z**
Cerebellar Vermis (4/5)	701	6.104	-3	-49	7
L Inferior Temporal Gyrus	326	4.913	-62	-37	-16

For all clusters, t > 3.10; p < 0.001 uncorrected; df = 40; minimum extent = 20 (due to conjunction); Table shows all local maxima separated by more than 20 mm. Regions were labeled using the HarvardOxford atlas and /or the AnatomyToolbox in parentheses; x,y,z =Montreal Neurological Institute (MNI) coordinates in the left-right, anterior-posterior, and inferior-superior dimensions, respectively.

Supplementary Figure 7: MID task trial structure

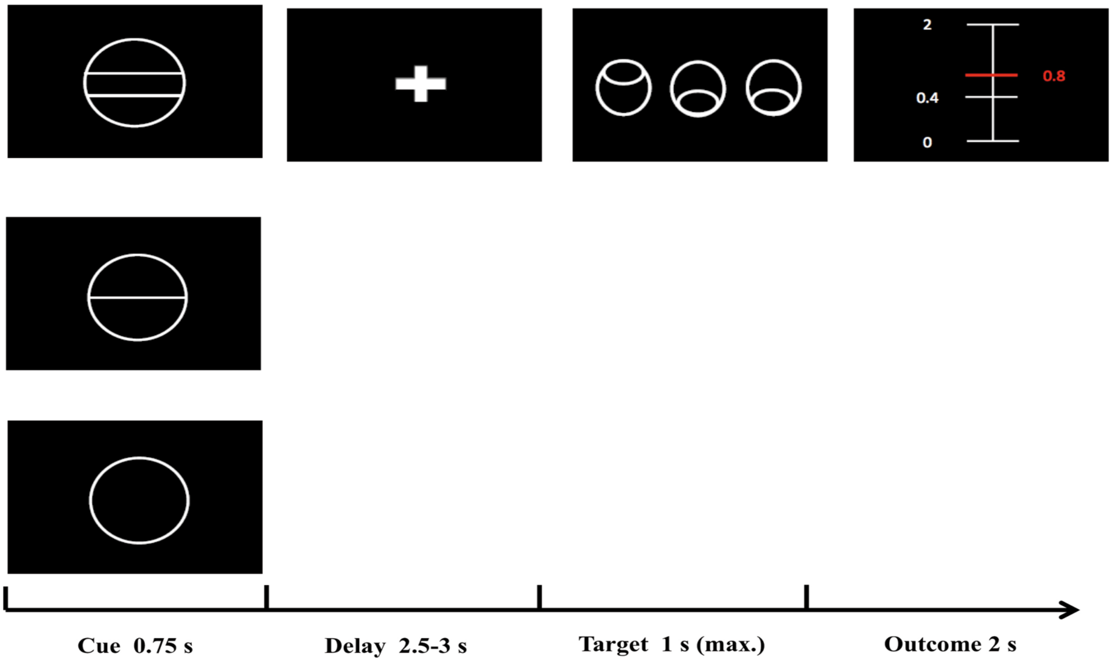

Supplementary Figure 7: Trial structure of the MID task. *First, one of three cues appeared. One cue was associated with large reward (ranging from 0 to 2.00 CHF), one cue with small reward (0 to 0.40 CHF) and one cue with no reward. After a delay of 2.5 to 3 s, participants had to identify an outlier from three circles by pressing one of three buttons as quickly as possible. Reward size depended on cue and response time.*

Supplementary Table 8: Strategies used

Supplementary Table 8: *Overview of the proposed strategies and how often these strategies had been used by the participants of the two groups.*

Proposed strategy	Veridical feedback Group (N=42)	Inverted feedback Group (N = 17)
**Family and friends**	27	3
**Food**	7	1
**Personal achievement**	9	1
**Romantic or sexual imagery**	39	8
**Leisure activities**	9	5
**Individual others**	-	-

Supplementary Note 3: Dynamic Causal Modelling analysis

Methods:

We analyzed task-dependent effective connectivity between SN/VTA and dlPFC during the second neurofeedback training run related to the upregulation of the dopaminergic midbrain. The model space comprised three model variants, namely a fully connected model, a top-down (dlPFC to SN/VTA) and a bottom-up (SN/VTA to dlPFC) model. Based on our findings from the PPI analysis, we we used IMAGINE_REWARD as modulatory effect on the connections between SN/VTA and dlPFC. Moreover, IMAGINE_REWARD and REST served as driving inputs on dlPFC.

We applied Bayesian model selection (BMS) followed by Bayesian parameter averaging (BPA). In addition, Bayesian model averaging (BMA) (Hoeting et al. 1999; Penny et al. 2010) was used to infer on the model structure (i.e., the significant connections between the ROIs), the connectivity parameters, and their modulations across the group. BMA provides an average over all parameter estimates within a model space weighted by their respective posterior probability. BMA can account for the uncertainty of the model structure and, at the same time, allows for inference on the connectivity parameters (Stephan et al. 2010).

In detail, for our DCM analysis, whitened and detrended time courses of dlPFC ($x = 40$, $y = 10$, $z = 38$) and SN/VTA ($x = -2$, $y = -16$, $z = -15$) regions were extracted for each participant using SPM12's volume of interest (VOI) extraction batch function. We extracted data from spheres within a radius of $r = 10$ mm, using a significance threshold of $p < 0.05$ uncorrected at the participant level and the first eigenvariate as summary statistic. One participant was excluded, as they did not exhibit significant activation in the search radius of both VOIs. Based on these time courses, DCM, as implemented in DCM12.5 (SPM12, Build 7771), was used to model the effective connectivity between these regions. All inputs (i.e., the time courses from our experimental conditions) were mean-centered. Finally, to investigate whether the modulatory effects of imagining reward are related to the transfer success of neurofeedback training on the individual level, we Spearman correlated them with midbrain DRT.

Results:

As result of Bayesian Model Selection, the fully connected model fitted the data of our study best (Supplementary Figure 8a). Within this model, the intrinsic connectivity between dlPFC and SN/VTA was positive (BMA posterior mean $A = + .097$) and weakly negative between SN/VTA and dlPFC (BMA posterior mean $A = - .004$). On top of this, self-regulation by neurofeedback training increased dlPFC to SN/VTA connectivity (BMA posterior mean $B = + .037$, see Supplementary Figure 8b). The results of BPA and BMA were comparable (Supplementary Table 9). Finally, the midbrain DRT was significantly negative correlated ($\rho = -.44$; $p = .003$) with the modulatory effect (Supplementary Figure 8c). Thus, successful self-regulation appears to benefit from some inhibitory modulation of SN/VTA by prefrontal cortex.

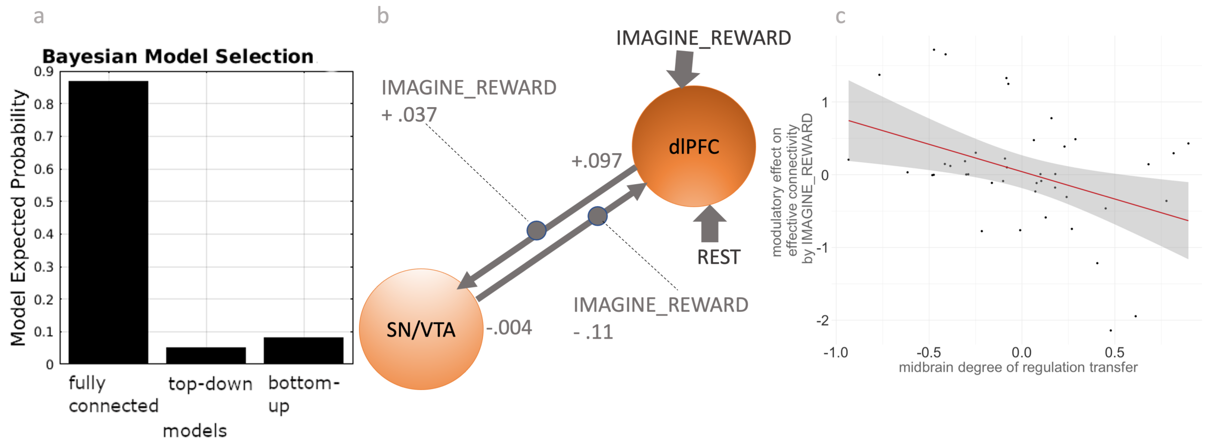

Supplementary Figure 8: DCM analysis reveals that neurofeedback training increases effective excitatory connectivity from dlPFC to SN/VTA while individual regulation success benefits from inhibitory connectivity*: (a) We tested three model variants, namely a fully connected, a top-down and a bottom-up model investigating effective connectivity between SN/VTA and dlPFC. Using Bayesian Model Selection, the fully connected model fits best to the data. (b) The employment of mental strategies during neurofeedback training increases the excitatory effective connectivity from dlPFC to SN/VTA. (C) Midbrain DRT is negatively correlated (ρ = -.44; p = .003) with the modulatory effect, i.e. dlPFC exerts an inhibitory effect for individuals with stronger transfer success. The grey shaded area identifies the 95 % confidence interval.*

Supplementary Table 9: *Result table of DCM modeling analysis*

**a**	**SN/VTA**	**dlPFC**
**SN**	- .05 (BPA)<br>- .05 (BMA)	+ .099 (BPA)<br>+ .097 (BMA)
**dlPFC**	- .007 (BPA)<br>- .004 (BMA)	- .066 (BPA)<br>- .068 (BMA)

**b_IMAGINE_REWARD**	**SN**	**dlPFC**	**ρ (with midbrain DRT)**
**SN**	0	+ .038 (BPA)<br>+ .037 (BMA)	ρ = -.44; p = .003 (BPA)<br>ρ = -.41; p = .007 (BMA)
**dlPFC**	- .12 (BPA)<br>- .11 (BMA)	0	

**c**	**IMAGINE_REWARD**	**REST**
**dlPFC**	+ .002<br>+. 0005	- .013 (BPA)<br>-.01 (BMA)

Discussion:

For both BPA and BMA analysis, the fully connected model outperformed the top-down or bottom-up only models. As expected from the literature, SN/VTA and dlPFC mutually modulate their connectivity. The neurofeedback training increases the positive effective connectivity from dlPFC to SN/VTA. Speculatively, the fact that this modulation correlates negatively with DRT may indicate that prefrontal cortex sculpts the general increase in connectivity to more successful strategies through inhibitory connectivity in successful individuals.

Supplementary Table 10: Confidence interval testing by bootstrapping

Supplementary Table 10: *We performed non-parametric bootstrapping using R scripts for all correlations presented throughout this manuscript to calculate confidence intervals at levels 2.5 % and 97.5 % of the distributions. The table summarizes the confidence intervals from the overall sample (Full sample) and a bootstrapping approach with 1000 subsamples. The confidence intervals using bootstrapping are close to the full sample intervals for all correlations reported in the main text and supplemental material. Therefore, this analysis supports the robustness of our findings.*

	**Full sample**		**Bootstrap (1000 samples)**	
**Analysis / ROI**	**2.5 %**	**97.5 %**	**2.5 %**	**97.5 %**
**Analysis Figure 2 (Correlation between midbrain and non-midbrain DRT in standard feedback group)**				
Intercept ROI Thalamus	-0.129	0.074	-0.121	0.069
Slope ROI Thalamus	0.574	1.301	0.574	1.256
Intercept ROI MFG	-0.114	0.095	-0.116	0.0927
Slope ROI MFG	0.3359	0.8162	0.4245	0.7654
Intercept	0.0233	0.2684	0.013	0.2706
Slope ROI ACC	0.4806	1.2034	0.482	1.1904
Intercept	-0.0974	0.1132	-0.1026	0.104
Slope ROI Temporal Cortex	0.2655	0.664	0.3252	0.6846
**Analysis Figure 3 (Correlation between midbrain and non-midbrain DRT in inverted feedback group)**				
Intercept ROI Amygdala	-0.0373	0.138	-0.0425	0.1178
Slope ROI Amygdala	0.5348	1.1567	0.5039	1.1111
**Analysis Figure 4 (Relation between midbrain temporal difference coding and dlPFC)**				
Intercept ROI dlPFC	-0.1579	0.0748	-0.1531	0.0761
Slope ROI dlPFC	-1.1782	-0.3293	-1.1893	-0.3025
**Analysis Figure 5 (Functional connectivity between dlPFC and SN/VTA**				
Intercept ROI SN/VTA	-0.0977	0.13	-0.092	0.12
Slope ROI SN/VTA	0.14	0.461	0.14	0.455
**Analysis Figure 6 (Reward-sensitivity in dlPFC correlates with successful SN/VTA self-regulation)**				
Intercept ROI dlPFC	-0.2324	0.1258	-0.225	0.1035
Slope ROI dlPFC	0.1674	1.121	0.3415	1.2012
**Analysis Supplementary Figure 2 (Positive correlation between the slope of SN/VTA signal change during neurofeedback training and midbrain DRT for the standard feedback group, but not for the control group)**				
Intercept standard NF group	-0.1747	0.1426	-0.17	0.153
Slope NF group	0.3294	0.7609	0.2563	0.8161
Intercept inverted NF group	-0.535	0.2412	-0.4621	0.1891
Slope inverted NF group	-0.8175	0.2294	-0.822	0.2421
**Analysis Supplementary Figure 8 (DCM analysis reveals that individual regulation success benefits from inhibitory connectivity)**				
Intercept modulatory effect	-0.1077	0.1371	-0.0993	0.1367
Slope modulatory effect	0.3789	-0.0626	-0.3169	-0.0999

Supplementary Note 4: Validation analysis of conditions *IMAGINE_REWARD* and *REST* as separate conditions

We performed a confirmatory analysis separating the two self-regulation conditions *IMAGINE_REWARD* and *REST* over the course of the study. Since the participants have been instructed to perform mental calculations during *REST*, which is an active task, these findings reveal that the results shown throughout the manuscript (*IMAGINE_REWARD-REST*) are not only driven by a decrease during *REST*.

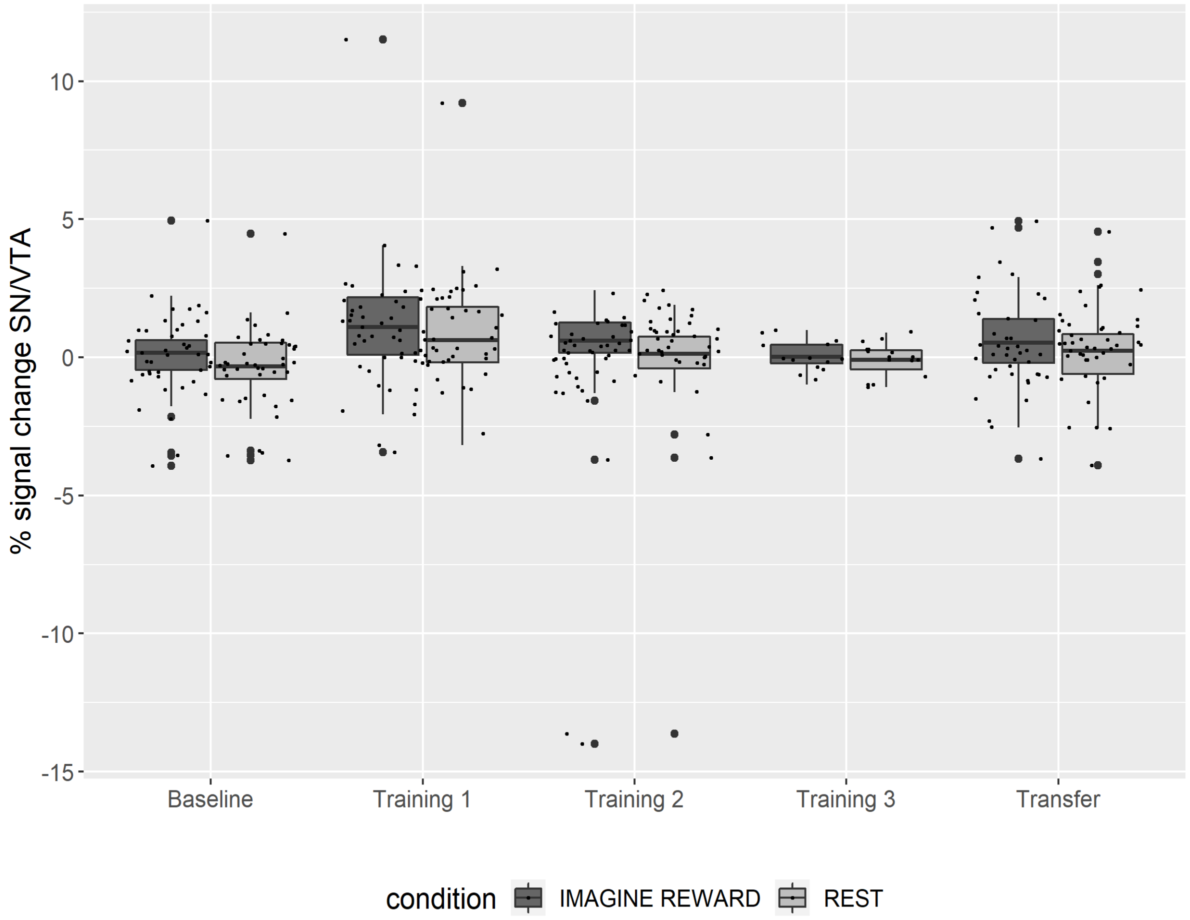

Supplementary Figure 9: BOLD signal change in the dopaminergic midbrain as separated by self-regulation conditions IMAGINE_RWARD and REST over the course of the study*: The BOLD signal change in the neurofeedback target regions SN/VTA is increasing while REST is not continuously decreasing over the course of the experiment (Note that Training 3 is based on Study 1 only). During the transfer run. The findings throughout this manuscript are based on the difference IMAGINE-REWARD-REST. This confirmatory analysis reveals that these finding are not driven by a decrease of REST over the course of the study. The vertical line in the boxplots goes through the box at the median and the boxes are drawn from the first to the third quartile. The error bars describe the standard error of the mean.*
