# Supplementary material for: Analysis of individual differences in neurofeedback training illuminates successful self-regulation of the dopaminergic midbrain
Source: Commun Biol. 2022 Aug 19;5:845. doi: 10.1038/s42003-022-03756-4 (PMC9391365; doi:10.1038/s42003-022-03756-4)
Supplement: Supplementary file 3 — Description of Additional Supplementary Files [file 42003_2022_3756_MOESM3_ESM.pdf]

Description of Additional Supplementary Files

File name: Supplementary Data 1
Description: : The source data behind the graphs in the paper and the supplemental material.
