# Supplementary material for: Analysis of individual differences in neurofeedback training illuminates successful self-regulation of the dopaminergic midbrain
Source: Commun Biol. 2022 Aug 19;5:845. doi: 10.1038/s42003-022-03756-4 (PMC9391365; doi:10.1038/s42003-022-03756-4)
Supplement: Supplementary file 5 — Reporting Summary [file 42003_2022_3756_MOESM5_ESM.pdf]

nature portfolio

Corresponding author(s): Dr. Lydia Hellrung

Last updated by author(s): Jun 28, 2022

Reporting Summary

Nature Portfolio wishes to improve the reproducibility of the work that we publish. This form provides structure for consistency and transparency in reporting. For further information on Nature Portfolio policies, see our Editorial Policies and the Editorial Policy Checklist.

Statistics

For all statistical analyses, confirm that the following items are present in the figure legend, table legend, main text, or Methods section.

n/a	Confirmed	
☐	☒	The exact sample size (*n*) for each experimental group/condition, given as a discrete number and unit of measurement
☐	☒	A statement on whether measurements were taken from distinct samples or whether the same sample was measured repeatedly
☐	☒	The statistical test(s) used AND whether they are one- or two-sided<br>*Only common tests should be described solely by name; describe more complex techniques in the Methods section.*
☐	☒	A description of all covariates tested
☐	☒	A description of any assumptions or corrections, such as tests of normality and adjustment for multiple comparisons
☐	☒	A full description of the statistical parameters including central tendency (e.g. means) or other basic estimates (e.g. regression coefficient) AND variation (e.g. standard deviation) or associated estimates of uncertainty (e.g. confidence intervals)
☐	☒	For null hypothesis testing, the test statistic (e.g. *F*, *t*, *r*) with confidence intervals, effect sizes, degrees of freedom and *P* value noted<br>*Give P values as exact values whenever suitable.*
☒	☐	For Bayesian analysis, information on the choice of priors and Markov chain Monte Carlo settings
☒	☐	For hierarchical and complex designs, identification of the appropriate level for tests and full reporting of outcomes
☐	☒	Estimates of effect sizes (e.g. Cohen's *d*, Pearson's *r*), indicating how they were calculated

Our web collection on statistics for biologists contains articles on many of the points above.

Software and code

Policy information about availability of computer code

Data collection	Data were collected in a Philips Achieva 3.0 T magnetic resonance (MR) scanner with an eight channel SENSE head coil (Philips, Best, The Netherlands) at the Laboratory for Social and Neural Systems Research (SNS), Zurich and the MR Center of the Psychiatric Hospital of the University of Zurich. MR-compatible electro- cardiogram (ECG), respiration and skin conductance (PowerLab 4/25 T and Chart v5.5.2, ADInstruments, Bella Vista, Australia) measurements were collected from each participant. Individual brain volumes were converted from Philips PAR/REC format to ANALYZEDRIN using software from Philips and then placed on a server in real time. A laptop running Turbo BrainVoyager v3.0 (TBV — Brain Innovation, Maastricht, The Netherlands) extracted the BOLD signal from these files, and redirected to provide visual feedback of neural activation using custom-made software on the same laptop with Visual Studio 2008 (Microsoft, Redmond, WA, USA). The subjects viewed the visual feedback through a mirror mounted on the head coil reflecting a back-projected display behind the bore.
Data analysis	- fMRI preprocessing: FSL 5, ANTs within FSL 5<br>- fMRI statistics: SPM 12 (v6906) within Matlab R2016b.<br>- fMRI figures were created using bspmview v.2016110844 and ggplot2 within R 3.4.1<br>- correlation results and plotting: R 3.4.1 with ggplot2<br>- custom-made analysis code is available in the repository https://github.com/lydiatgit/NFLearning_SNVTA_PublicRepo as stated in the data availibility statement

For manuscripts utilizing custom algorithms or software that are central to the research but not yet described in published literature, software must be made available to editors and reviewers. We strongly encourage code deposition in a community repository (e.g. GitHub). See the Nature Portfolio guidelines for submitting code & software for further information.

Data

Policy information about availability of data

All manuscripts must include a data availability statement. This statement should provide the following information, where applicable:

- Accession codes, unique identifiers, or web links for publicly available datasets
- A description of any restrictions on data availability
- For clinical datasets or third party data, please ensure that the statement adheres to our policy

Second level statistical map data supporting the findings of this study are available at https://identifiers.org/neurovault.collection:12684. All other source data, such as data extractions from regions of interest and mask files, for this paper and the Supplemental Material are provided with this paper. A reporting summary for this Article is available as a Supplementary Information file.

Field-specific reporting

Please select the one below that is the best fit for your research. If you are not sure, read the appropriate sections before making your selection.

☒ Life sciences ☐ Behavioural & social sciences ☐ Ecological, evolutionary & environmental sciences

For a reference copy of the document with all sections, see nature.com/documents/nr-reporting-summary-flat.pdf

Life sciences study design

All studies must disclose on these points even when the disclosure is negative.

Sample size	In this study, we combined data from two previous studies. Therefore, we used all available data, which in this case were fifty-nine participants.
Data exclusions	Here and in the previous studies, two participants were excluded, one due to artifacts in functional images and one because of difficulties with the head coil during the experiment.
Replication	We analyzed the data from the two previous studies with a new approach to investigate the mechanisms underlying successful SN/VTA self-regulation. As such an analysis has not been done before, we did not aim to replicate findings. Nonetheless, our findings are in line with animal literature and simulation data from other researchers.
Randomization	Invited participants were randomly assigned to veridical or false feedback groups.
Blinding	The experimenters have not been blinded because the setup of the control group required loading of data from a participant of the veridical feedback group. Regardless of group, identical instructions were given in written form and experimenter-subject interactions were minimal.

Reporting for specific materials, systems and methods

We require information from authors about some types of materials, experimental systems and methods used in many studies. Here, indicate whether each material, system or method listed is relevant to your study. If you are not sure if a list item applies to your research, read the appropriate section before selecting a response.

Materials & experimental systems

n/a	Involved in the study	
☒	☐	Antibodies
☒	☐	Eukaryotic cell lines
☒	☐	Palaeontology and archaeology
☒	☐	Animals and other organisms
☐	☒	Human research participants
☒	☐	Clinical data
☒	☐	Dual use research of concern

Methods

n/a	Involved in the study	
☒	☐	ChIP-seq
☒	☐	Flow cytometry
☐	☒	MRI-based neuroimaging

Human research participants

Policy information about studies involving human research participants

Population characteristics	Fifty-nine right-handed participants (45 males, average age 28.25±5.25 years)
Recruitment	Study 1 recruited healthy male, non-smoking participants from a departmental database of university students. In Study 2, healthy participants were recruited via online advertisement to match an inpatient group of cocaine addiction patients with

regard to sex, age and nicotine consumption. Exclusion criteria were clinically significant somatic diseases, head injury or neurological disorders, family history of schizophrenia or bipolar disorder, and use of prescription drugs affecting the central nervous system. Additional exclusion criteria for both study groups were MRI ineligibility due to non-removable ferromagnetic objects in the body, claustrophobia, or pregnancy.

Ethics oversight	The ethics committee of the canton of Zurich approved these studies in accordance with the Human Subjects Guidelines of the Declaration of Helsinki.

Note that full information on the approval of the study protocol must also be provided in the manuscript.

Magnetic resonance imaging

Experimental design

Design type	block design neurofeedback task
Design specifications	4 consecutive runs, each containing 9 blocks of two regulation conditions (18 blocks per run), first and last run without feedback presentation, second and third run with feedback presentation
Behavioral performance measures	1) Strategies used during neurofeedback training - comparison between intervention and control group<br>2) Personality measures from questionnaires - comparison of scores between intervention and control group<br>3) Reward sensitivity measure from independent MID task - correlation with neurofeedback performance

Acquisition

Imaging type(s)	functional, structural
Field strength	3.0
Sequence & imaging parameters	Structural: (Study1) gradient echo T1-weighted sequence in 301 sagittal plane slices of 250 × 250 mm2 resulting in 1.1 mm3 voxels; (Study2) spin-echo T2-weighted sequence with 70 sagittal plane slices of 230 × 184 mm2 resulting in 0.57 × 0.72 × 2 mm3 voxel size<br>Functional: gradient echo T2*-weighted whole brain echo-planar image sequence in both studies. The in-plane resolution was 2 × 2 mm2, 3 mm slice thickness and 1.1 mm gap width over a field of view of 220 × 220 mm2, a TR/TE of 2000/35 ms and a flip angle of 82°. Slices were aligned with the anterior–posterior commissure and then tilted by 15°
Area of acquisition	whole-brain

Diffusion MRI ☐ Used ☒ Not used

Preprocessing

Preprocessing software	FSL 5 (FMRIB Software Library, Analysis Group, FMRIB, Oxford, http://fsl.fmrib.ox.ac.uk). Furthermore, data were bias-field corrected using ANTs (Advanced Normalization Tools; http://stnava.github.io/ANTs).
Normalization	The data were normalized to standard Montreal Imaging Institute (MNI) space using ANTs in combination with a custom scanner-specific EPI-template resulting in a 1.5 mm3 isotropic resolution
Normalization template	MNI152
Noise and artifact removal	We used an image-based correction to account for physiological artifacts in all participants. Because physiological artifacts are most prominently present in CSF and white matter due to the absence of BOLD effects, pulsations of the ventricles, and proximity to the large brain arteries (e.g., circle of Willis), we decided to use an established preprocessing procedure based on a principal component analysis (PCA) approach. Specifically, we calculated the global mean and the first six components of a temporal principal component analysis on the cerebrospinal fluid and white matter signal. These six components were used as noise regressors in the first level statistics in addition to the six motion parameters.
Volume censoring	Five dummy-image excitations were performed and discarded before functional image acquisition started, otherwise no censoring was applied.

Statistical modeling & inference

Model type and settings	Mass-univariate random effects models on first level, correlation with individual degree of regulation transfer on second level
Effect(s) tested	The GLMs contained two conditions - IMAGINE_REWARD and REST. We tested for whole brain transfer effects (last run - first run) based on the difference between these two conditions for 1) an intervention group that received veridical feedback and 2) a control group that received inverted feedback. Furthermore, we tested for the effect of a temporal difference parametric modulator during the training runs of the neurofeedback task (second and third run). Finally, we tested for the correlation of these effects with the degree of regulation transfer (midbrain DRT).

Specify type of analysis: ☐ Whole brain ☐ ROI-based ☒ Both

Anatomical location(s) | Substantia Nigra/ Ventral Tegemental Area: extracted from probabilistic atlas mask by Murty et al. (2014)

Statistic type for inference
(See Eklund et al. 2016) | Cluster -wise, local maxima separated by more than 20 mm; $p < 0.05$ FWE-corrected on cluster level; df = 40.

Correction | $p < 0.05$ FWE-corrected on cluster level

Models & analysis

n/a	Involved in the study
☐	☒ Functional and/or effective connectivity
☒	☐ Graph analysis
☒	☐ Multivariate modeling or predictive analysis

Functional and/or effective connectivity

1) PPI: we investigated the functional impact of temporal difference coding in dlPFC on SN/VTA activity using a psychophysiological interaction analysis with the gPPI v13 Toolbox. We added activity from the dlPFC seed region as physiological regressor to the original GLM and interacted it with both condition regressors to form interaction regressors.

2) DCM: We investigated task-dependent effective connectivity between SN/VTA and dlPFC during the second neurofeedback training run related to the upregulation of the dopaminergic midbrain. The model space comprised three model variants: a fully connected model, a top-down (dlPFC to SN/VTA) and a bottom-up (SN/VTA to dlPFC) model.
